# Prehospital 12-lead ECG and outcomes in acute coronary syndrome

**DOI:** 10.1136/heartjnl-2025-325780

**Published:** 2025-09-10

**Authors:** Timothy John Driscoll, Sarah Black, Glenn Davies, Chris P Gale, Lucia Gavalova, Mary Halter, Chelsey Hughes, Scott Munro, Nigel Rees, Andy Rosser, Helen Snooks, Alan Watkins, Clive Weston, Tom Quinn

**Affiliations:** 1 Swansea University Medical School, Swansea, UK; 2 South Western Ambulance Service NHS Foundation Trust, Exeter, UK; 3 Patient and Public Representative, London, UK; 4 Division of Epidemiology and Biostatistics, University of Leeds, Leeds, UK; 5 Kingston University, London, UK; 6 Welsh Ambulance Services University NHS Trust, Cwmbran, UK; 7 South East Coast Ambulance Service NHS Foundation Trust, Crawley, UK; 8 School of Health Sciences, University of Surrey, Guildford, UK; 9 West Midlands Ambulance Service University NHS Foundation Trust, Brierley Hill, UK; 10 Glangwili General Hospital, Carmarthen, UK

**Keywords:** Acute Coronary Syndrome, Electronic Health Records

## Abstract

**Importance/background:**

The 12-lead ECG is recommended in clinical guidelines for prehospital assessment of patients with suspected acute coronary syndrome (ACS) presenting to Emergency Medical Services (EMS).

**Objectives:**

To determine prehospital ECG (PHECG) utilisation since UK national rollout of primary percutaneous coronary intervention, and whether this is associated with clinical outcomes in patients with ACS.

**Design:**

Population-based, linked cohort study using Myocardial Ischaemia National Audit Project data from 1 January 2010 to 31 December 2017, related to patients with ACS conveyed by the EMS to hospital in England and Wales.

**Exposure:**

PHECG administration.

**Outcomes:**

Proportion of patients where PHECG was recorded, 30-day and 1 year all-cause mortality, use of reperfusion.

**Results:**

Of 330 713 eligible patients transferred by EMS, 263 420 patients (79.7%) had PHECG recorded, steadily increasing from 74.2% in 2010 to 85.0% in 2017. Patients who received PHECG were generally younger than those who did not (median age: 70 years vs 75 years), less likely to be female (32.8% vs 41.9%) or to have comorbidities such as diabetes (20.8% vs 24.7%) or peripheral vascular disease (4.1% vs 4.8%). Patients who received PHECG had lower mortality at 30 days (7.1% vs 10.9%), with adjusted OR 0.77 (95% CI 0.75 to 0.80), and at 1 year (14.2% vs 23.2%), with adjusted OR 0.69 (95% CI 0.68 to 0.71). Adjustment accommodated demographic characteristics, comorbidities and medical history. Reperfusion was more frequent in patients with ST-elevation myocardial infarction (STEMI) receiving PHECG (84.5% vs 54.7%) with adjusted OR 4.37 (95% CI 4.20 to 4.54), with similar adjustment.

**Conclusions:**

Use of PHECG by EMS for patients with ACS is associated with lower short-term mortality and higher odds of receiving reperfusion for STEMI patients. Administration of PHECG increased steadily over time, but at the end of the study, still 15% of eligible patients did not receive a PHECG.

**WHAT IS ALREADY KNOWN ON THIS TOPIC**

- Prehospital ECG (PHECG) is associated with better survival in patients with acute coronary syndrome (ACS) but is potentially underused.

**WHAT THIS STUDY ADDS**

- This study updates previous research and examines whether the proportion of patients that received PHECG changed between 2010 and 2017.

**HOW THIS STUDY MIGHT AFFECT RESEARCH, PRACTICE OR POLICY**

- Findings provide evidence on PHECG administration and factors associated with its use for patients with ACS since the introduction of primary percutaneous coronary intervention.

## Introduction

 International guidelines and quality indicators for emergency cardiovascular care recommend the performance of a 12-lead ECG by Emergency Medical Services (EMS) personnel during assessment of patients with suspected acute coronary syndrome (ACS).^1 2^ Information gained from a prehospital 12-lead ECG (PHECG) informs decision-making in three components of immediate care: targeted prehospital treatment, transportation to an appropriate receiving hospital, and activation of the receiving cardiac catheter laboratory when ST-segment elevation myocardial infarction (STEMI) is identified.^3^

Research has focused on the association of PHECG with process-of-care quality descriptors, such as ‘call-to-reperfusion’ time.^4^ A systematic review and meta-analysis confirmed the association of PHECG with improved clinical outcomes.^5^ However, PHECG was underused, particularly in older patients, women and people with comorbidities. Our previous work assessing use and impact of PHECG was undertaken in England and Wales when fibrinolytic therapy was the principal reperfusion strategy for STEMI.^6^ Primary percutaneous coronary intervention (pPCI) became widely available in England and Wales in 2009, with 95% of eligible patients receiving pPCI by 2012/2013.^7^ This change in practice necessitated changes in EMS care of STEMI patients, with direct transportation to pPCI capable hospitals replacing prior strategies of either prehospital or in-hospital fibrinolytic treatment.

The purpose of this study was to assess PHECG use, and related outcomes, since the introduction of pPCI. Specifically, our three ‘research questions’ were: whether the proportion of eligible patients receiving PHECG changed since the national rollout of pPCI networks; whether patients who received PHECG differed from those who did not; and how patients who did and did not receive PHECG differed with respect to prehospital times, reperfusion strategy, and 30-day and 1-year mortality.

## Methods

The study protocol has been published previously.^8^ We performed a population-based, linked cohort study using 2010–2017 UK national heart attack data from the Myocardial Ischaemia National Audit Project (MINAP), a comprehensive registry of ACS hospitalisations mandated by the Department of Health and Social Care, to update evidence on care and outcomes for patients eligible for PHECG. At the time of the study, MINAP provided patient demographic and clinical details across 122 data items.^9^

### Patient and public involvement

Three patient and public representative has been involved since study inception. They played a role in the development of relevant research questions, the study proposal and funding application, oversight activities, review and interpretation of results and dissemination of findings.

### Inclusion/exclusion criteria

Patients aged 18 years or older when attended by EMS between 1 January 2010 and 31 December 2017 and admitted with ACS to a hospital in England and Wales participating in MINAP were included. For patients with multiple admissions, only the first ACS event in this period was included. Analysis was by final diagnosis recorded.

### Data collection

MINAP data are collected prospectively using a secure electronic system, encrypted and transferred online to a central database which protects patient identity. Data were anonymised and imported into the Secure eResearch Platform UK trusted research environment and deterministically linked with Office for National Statistics (ONS, civil registry) mortality data from NHS England. The analysis team did not have access to data that would allow records to be linked back to named individuals.

### Statistical analysis

To explore any change in the proportion of patients receiving PHECG over time, we tabulated the proportion of patients receiving PHECG and conducted a linear regression analysis. Data are presented overall and by EMS.

For our second and third questions, we compared demographics, comorbidities and medical histories prior to the index ACS event, incorporating these core factors: age (dichotomised as 18–74 years/75+ years); sex; previous validated episode of acute myocardial infarction (MI); previous coronary artery bypass graft (CABG); diabetes and previous chronic heart failure (CHF). Factors were selected for consistency with previous work and are listed with each table.^6^ ORs, with 95% CI, are presented throughout, being more informative than p values, which were generally highly significant even for small absolute differences due to cohort size.

We used logistic regression to investigate any association between receipt of PHECG and prespecified demographic and clinical factors. The dependent variable was receipt of PHECG. In addition to the core factors, we also adjusted for ethnicity (Caucasian/Asian/other); hypertension; peripheral vascular disease (PVD); whether the patient currently smokes; dyslipidaemia; prior PCI; prior stroke; chronic kidney disease (CKD); prior angina; and asthma/chronic obstructive pulmonary disease (COPD).

Prehospital haemodynamic data are not available in MINAP. Instead, we conducted unadjusted comparisons of heart rate at, and first systolic blood pressure after, hospital admission using Mann-Whitney U tests. As post-PHECG data, these were not incorporated into the logistic regression investigating use of PHECG.

We used logistic regression to investigate any association between 30-day and 1-year mortality and PHECG use. In addition to the core factors, we adjusted for ethnicity (as above), receipt of aspirin, raised cardiac markers, CKD and prior stroke/cerebrovascular disease. We used Cox regression models to investigate differences in the time from call for help to arrival at hospital, and from ambulance arrival to arrival at hospital, between patients who did, and did not, receive PHECG. In addition to the core factors above, we adjusted for ethnicity (as above), CKD and prior stroke. The proportional hazards assumption was verified using Schoenfeld residual plots and log(-log(survival)) plots. Time intervals were right-censored at 1 day.

For STEMI patients, we used logistic regression to investigate any association between provision of reperfusion therapy and PHECG. The dependent variable was receipt of reperfusion. We adjusted for core factors and incident year only.

For STEMI patients receiving reperfusion, we used Cox regression to investigate whether there was a difference in time to reperfusion treatment associated with PHECG. The dependent variable was time to reperfusion (minutes) from call for help, from ambulance arrival and from hospital door. We adjusted for core factors, ethnicity (as above), CKD and prior stroke. The proportional hazards assumption was verified using Schoenfeld residual plots and log(-log(survival)) plots. Time intervals were right-censored at 1 day. We also used logistic regression, adjusted as per the Cox regression, to investigate whether PHECG use was associated with receipt of reperfusion within 90 min of the call for help. The dependent variable was receipt of reperfusion within 90 min.

### Treatment of missing data

We used multiple imputation to mitigate against bias due to missing data, generating five sets of imputations using Fully Conditional Specification using the full MINAP dataset. The imputation model included the following variables, chosen for consistency with the previous work^6^: core factors as above; STEMI; conveyance by EMS; ethnicity (as above); prior angina; hypertension; dyslipidaemia; PVD; prior stroke/cerebrovascular disease; asthma/COPD; CKD; whether seen by a cardiologist; prior PCI; current smoker; mini-Global Registry of Acute Coronary Events^10^ risk score tercile; Index of Multiple Deprivation quartile; systolic blood pressure quartile; heart rate at admission quartile; whether a delay in treatment was recorded in MINAP; raised cardiac markers. Imputed data were used for all analyses, except use of PHECG over time, for which data were complete.

### Statistical software

Data were analysed using SPSS, V.26.

## Results

**Figure 1:**
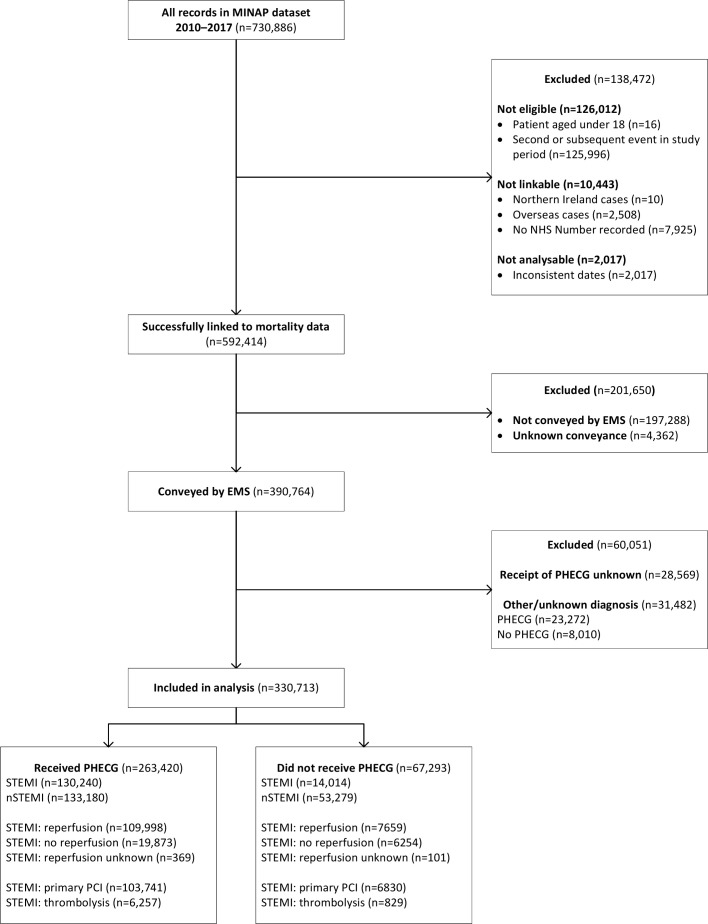
Flow diagram. EMS, Emergency Medical Service; MINAP, Myocardial Ischaemia National Audit Project; nSTEMI, non-STEMI; PCI, percutaneous coronary intervention; PHECG, prehospital ECG; STEMI, ST-elevation myocardial infarction.

The MINAP registry (2010–2017) included 730 886 ACS records (figure 1). We excluded 138 472 records (18.9%) because the patient or event was ineligible, we could not link MINAP and ONS data, or data were otherwise not analysable. Most of these exclusions (125 996 records) were the second or subsequent ACS event for that patient within the study period. The remaining 592 414 records were linked to mortality data with a further 201 650 excluded where not conveyed by EMS, or where mode of conveyance was unknown.

Of the remaining 390 764 patients conveyed by EMS, 60 051 were excluded because whether they received PHECG (28 569 patients) or final diagnosis (31 482) was unknown. This resulted in an analysis cohort of 330 713 patients.

Overall, four-fifths (79.7%) of patients received PHECG. Table 1 shows overall PHECG use increased from 74.2% in 2010 to 85.0% in 2017, an annual increase of 1.59%-points. Although more patients conveyed by each EMS received PHECG in 2017 vs 2010, the proportion varied by EMS, and in some cases fell year-on-year.

**Table 1:** Proportion of patients receiving PHECG per year

		Received PHECG								Regression coefficient (95% CI)
		2010	2011	2012	2013	2014	2015	2016	2017	
EMS 1	n/N	3348/4104	3015/3663	3209/3903	3328/3858	2765/3170	1579/1791	1550/1747	1727/2038	0.87%pt/yr (0.09% to 1.64%)
	%	81.6%	82.3%	82.2%	86.3%	87.2%	88.2%	88.7%	84.7%	
EMS 2	n/N	3278/4215	3383/4368	3038/3804	2920/3586	2677/3274	2913/3427	3133/3629	3578/4032	1.63%pt/yr (1.31% to 1.95%)
	%	77.8%	77.4%	79.9%	81.4%	81.8%	85.0%	86.3%	88.7%	
EMS 3	n/N	2341/3036	2148/2737	2227/2876	2341/2933	2300/2752	2454/2778	2572/3006	2803/3199	1.73%pt/yr (1.00% to 2.46%)
	%	77.1%	78.5%	77.4%	79.8%	83.6%	88.3%	85.6%	87.6%	
EMS 4	n/N	1856/2773	1950/2775	1848/2555	1693/2274	1736/2214	1579/2014	1458/1858	1698/1889	2.67%pt/yr (1.65% to 3.68%)
	%	66.9%	70.3%	72.3%	74.5%	78.4%	78.4%	78.5%	89.9%	
EMS 5	n/N	3929/6003	3920/6039	4007/5824	3316/4909	3172/4458	3186/4201	3455/4237	3958/4922	2.53%pt/yr (1.67% to 3.40%)
	%	65.5%	64.9%	68.8%	67.5%	71.2%	75.8%	81.5%	80.4%	
EMS 6	n/N	2051/2806	2037/2581	2097/2497	1942/2364	1955/2307	2081/2424	1654/1846	1872/2092	2.10%pt/yr (1.31% to 2.89%)
	%	73.1%	78.9%	84.0%	82.1%	84.7%	85.8%	89.6%	89.5%	
EMS 7	n/N	2371/2961	2421/3007	2503/2975	2389/2855	2348/2574	2307/2478	2507/2725	2668/2938	1.99%pt/yr (1.04% to 2.94%)
	%	80.1%	80.5%	84.1%	83.7%	91.2%	93.1%	92.0%	90.8%	
EMS 8	n/N	3036/3862	3429/4005	3523/4034	3376/3867	3329/3700	3250/3601	3495/3953	3899/4423	1.10%pt/yr (0.07% to 2.13%)
	%	78.6%	85.6%	87.3%	87.3%	90.0%	90.3%	88.4%	88.2%	
EMS 9	n/N	1460/1923	1719/2147	1729/2113	1548/1902	1467/1635	1375/1531	1401/1613	1463/1609	2.04%pt/yr (1.09% to 2.99%)
	%	75.9%	80.1%	81.8%	81.4%	89.7%	89.8%	86.9%	90.9%	
EMS 10	n/N	1681/2293	2369/2853	2742/3060	2615/2840	2679/2893	2772/2942	2450/2572	2632/2745	2.78%pt/yr (1.30% to 4.26%)
	%	73.3%	83.0%	89.6%	92.1%	92.6%	94.2%	95.3%	95.9%	
EMS 11	n/N	3192/4189	3359/4005	3223/3825	3086/3580	3123/3567	3061/3519	2843/3193	2924/3218	1.64%pt/yr (0.87% to 2.42%)
	%	76.2%	83.9%	84.3%	86.2%	87.6%	87.0%	89.0%	90.9%	
EMS 12	n/N	139/176	86/123	100/130	129/158	104/115	71/85	68/83	125/145	1.66%pt/yr (−0.24% to 3.56%)
	%	79.0%	69.9%	76.9%	81.6%	90.4%	83.5%	81.9%	86.2%	
England and Wales	n/N	29 952/40 378	32 297/42 342	34 032/44 066	32 822/42 111	33 174/41 322	33 293/40 274	32 932/39 126	34 918/41 094	1.59%pt/yr (1.38% to 1.81%)
	%	74.2%	76.3%	77.2%	77.9%	80.3%	82.7%	84.2%	85.0%	

Individual cells may not sum to totals due to incidents where the conveying EMS is not known.

EMS, Emergency Medical Service; PHECG, prehospital ECG; %pt/yr, percentage points per year.

Patients receiving PHECG were typically younger (median age 70 years (IQR 59–80) vs 75 (64–84)), less frequently female and less likely to have diabetes, hypertension or PVD (table 2). They were more likely to be current smokers, have dyslipidaemia or previous PCI, but less likely to have other medical history recorded.

**Table 2:** Comparison of patients conveyed by EMS who do and who do not receive a PHECG

	PHECG (n=263 420)		No PHECG (n=67 293)		Adjusted OR	95% CI
	n	%	n	%		
Core factors						
Age (median, quartiles)	70	59–80	75	64–84	–	–
Aged >75 years	101 537/263 278 Missing=142	38.6%	34 566/67 202 Missing=91	51.4%	0.73	0.72 to 0.75
Female	86 030/261 923 Missing=1497	32.8%	28 122/67 050 Missing=243	41.9%	0.76	0.75 to 0.78
Prior myocardial infarction	46 058/250 031 Missing=13 389	18.4%	13 692/64 818 Missing=2475	21.1%	0.92	0.90 to 0.95
Prior coronary artery bypass surgery	14 702/248 376 Missing=15 044	5.9%	4276/64 388 Missing=2905	6.6%	1.01	0.97 to 1.05
Prior heart failure	12 277/248 234 Missing=15 186	4.9%	5114/64 358 Missing=2935	7.9%	0.80	0.77 to 0.83
Other factors						
White	219 975/238 006 Missing=25 414	92.4%	58 034/62 321 Missing=4972	93.1%	(reference category)	–
Asian	16 241/238 006 Missing=25 414	6.8%	3644/62 321 Missing=4972	5.8%	1.05	1.00 to 1.10
Other ethnic group	1790/238 006 Missing=25 414	0.8%	643/62 321 Missing=4972	1.0%	0.98	0.93 to 1.04
Hypertension	124 619/250 428 Missing=12 992	49.8%	34 382/65 030 Missing=2263	52.9%	1.00	0.99 to 1.03
Peripheral vascular disease	10 103/247 441 Missing=15 979	4.1%	3088/63 889 Missing=3404	4.8%	0.95	0.91 to 0.99
Diabetes	53 205/255 497 Missing=7923	20.8%	16 213/65 715 Missing=1578	24.7%	0.86	0.84 to 0.88
Current smoker	72 611/246 332 Missing=17 088	29.5%	13 891/62 815 Missing=4478	22.1%	1.22	1.19 to 1.24
Dyslipidaemia	78 369/245 893 Missing=17 527	31.9%	19 275/63 681 Missing=3612	30.3%	1.10	1.08 to 1.12
Prior percutaneous coronary intervention	22 305/248 289 Missing=15 131	9.0%	5160/64 258 Missing=3035	8.0%	1.22	1.18 to 1.27
Prior stroke	20 205/248 143 Missing=15 277	8.1%	7311/64 384 Missing=2909	11.4%	0.83	0.81 to 0.86
Chronic kidney disease	13 953/247 367 Missing=16 053	5.6%	5758/64 300 Missing=2993	9.0%	0.78	0.76 to 0.81
Prior angina	52 900/248 022 Missing=15 398	21.3%	16 351/64 384 Missing=2909	25.4%	0.91	0.88 to 0.93
Asthma/chronic obstructive pulmonary disease	37 483/248 653 Missing=14 767	15.1%	12 412/64 556 Missing=2737	19.2%	0.80	0.79 to 0.82

ORs are adjusted for PHECG use, age (greater than 75 vs not), sex, ethnicity (Caucasian vs Asian vs other ethnic groups), receipt of aspirin, raised cardiac markers, chronic kidney disease, diabetes, prior stroke/cerebrovascular disease and prior CHF. Individual categories may not sum to the total due to missing data.

CHF, chronic heart failure; EMS, Emergency Medical Services; PHECG, prehospital ECG.

A multivariate model to account for mutual confounding found that being older than 75 years, female and recording of PVD, diabetes and history of prior MI, heart failure, stroke, CKD, angina and asthma or COPD were all associated with lower odds of receiving PHECG, while current smokers, dyslipidaemia and prior PCI were associated with higher odds. PHECG was more common in patients with a final diagnosis of STEMI (90.3%) than those with nSTEMI (71.4%).

Patients receiving PHECG had a lower median heart rate (77/min (IQR 65–90) vs 80 (68–96), Mann-Whitney U test statistic −46.21, p<0.01) and systolic blood pressure (135 mm Hg (117–154) vs 137 (119–156), Mann-Whitney U-test statistic −15.45, p<0.01) at admission than those without PHECG.

Patients with PHECG recorded had lower mortality than those without PHECG at 30 days (7.1% vs 10.9%; adjusted OR (aOR) 0.77; 95% CI 0.75 to 0.80) and 1 year (14.2% vs 23.2%; aOR 0.69; 95% CI 0.68 to 0.71) (table 3). A mortality benefit was observed for STEMI patients alone, nSTEMI alone and for STEMI patients who received reperfusion.

**Table 3:** 30-day and 1-year mortality by receipt of PHECG

	PHECG		No PHECG		Adjusted OR	95% CI
Total population	(n=2 63 420)	%	(n=67 293)	%	–	–
30 days	18 591	7.1%	7334	10.9%	0.77	0.75 to 0.80
1 year	37 535	14.2%	15 623	23.2%	0.69	0.68 to 0.71
nSTEMI patients	(n=1 33 180)	%	(n=53 279)	%	–	–
30 days	8151	6.1%	5109	9.6%	0.68	0.65 to 0.70
1 year	21 767	16.3%	12 425	23.3%	0.69	0.68 to 0.71
STEMI patients	(n=1 30 240)	%	(n=14 014)	%	–	–
30 days	10 440	8.0%	2225	15.9%	0.59	0.56 to 0.62
1 year	15 768	12.1%	3198	22.8%	0.59	0.56 to 0.61
STEMI patients not receiving reperfusion	(n=19 873)	%	(n=6254)	%	–	–
30 days	4235	21.3%	1547	24.7%	0.91	0.85 to 0.98
1 year	6141	30.9%	2276	36.4%	0.87	0.81 to 0.93
STEMI patients receiving reperfusion therapy	(n=1 09 998)	%	(n=7659)	%	–	–
30 days	6144	5.6%	656	8.6%	0.65	0.59 to 0.71
1 year	9541	8.7%	889	11.6%	0.74	0.68 to 0.80
STEMI patients receiving primary PCI	(n=1 03 741)	%	(n=6352)	%	–	–
30 days	5478	5.3%	475	7.0%	0.74	0.67 to 0.83
1 year	8710	8.4%	672	9.8%	0.83	0.76 to 0.91
STEMI patients receiving thrombolysis	(n=6257)	%	(n=829)	%	–	–
30 days	630	10.1%	829	21.5%	0.49	0.40 to 0.60
1 year	823	13.2%	217	26.2%	0.50	0.41 to 0.61
Second/subsequent events included	(n=3 00 207)	%	(n=77 584)	%	–	–
30 days	21 445	7.1%	8372	10.8%	0.75	0.72 to 0.77
1 year	45 789	15.3%	18 586	24.0%	0.70	0.69 to 0.72

ORs are adjusted for age (greater than 75 vs not), sex, ethnicity (Caucasian vs Asian vs other ethnic groups), receipt of aspirin, raised cardiac markers, chronic kidney disease, diabetes, prior stroke/cerebrovascular disease and prior CHF. ‘Second/subsequent events’ refers to any ACS event where the patient had a previous ACS event recorded in MINAP during the study period.

ACS, acute coronary syndrome; CHF, chronic heart failure; MINAP, Myocardial Ischaemia National Audit Project; nSTEMI, non-STEMI; PCI, percutaneous coronary intervention; PHECG, prehospital ECG; STEMI, ST-elevation myocardial infarction.

The median time from call for help to arrival at hospital was 3 min longer for patients with PHECG than those without (65 min (IQR 51–84) vs 62 min (IQR 47–85)). However, adjusted HRs marginally favour PHECG (1.04; 95% CI 1.03 to 1.05), suggesting some of the difference may be explained by other factors (online supplemental figure 1).

The median time from ambulance arrival to hospital admission was 5 min longer for patients with PHECG than those without (50 min (IQR 38–66) vs 45 min (IQR 33–65)). Adjusted HRs suggest patients without PHECG spend less time under EMS care (adjusted HR 0.92, 95% CI 0.91 to 0.93).

**Figure 2:**
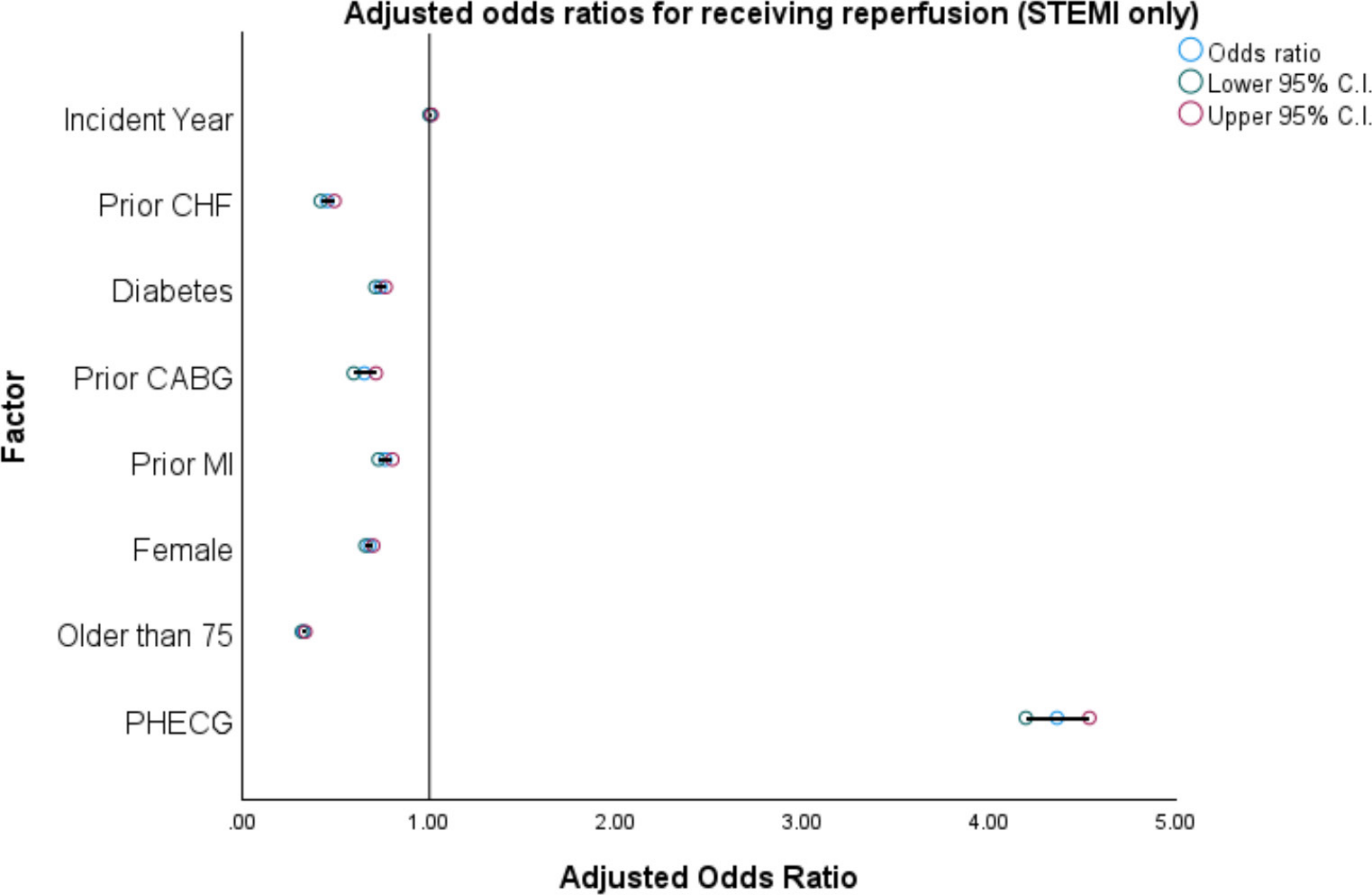
ORs for receipt of reperfusion in patients with STEMI. CABG, coronary artery bypass graft; CHF, chronic heart failure; MI, myocardial infarction; PHECG, prehospital ECG; STEMI, ST-elevation MI.

STEMI patients who received PHECG were more likely to receive reperfusion than those who did not after adjusting for confounding factors (aOR 4.37, 95% CI 4.20 to 4.54) (figure 2; online supplemental table 1). Reperfusion was less likely in patients aged 75+, females, with diabetes or prior MI, CABG or CHF. There was no association between incident year and likelihood of reperfusion. While pPCI was more common than thrombolysis for patients with, and without, PHECG, a higher proportion of patients with PHECG received pPCI (103 741/109 998 (94.3%) vs 6830/7,659 (89.2%), aOR 1.30, 95% CI 1.20 to 1.40).

For STEMI patients receiving reperfusion, the median time from ambulance arrival to reperfusion was 6 min shorter for patients with PHECG than without (97 min (IQR 78–120) vs 103 min (IQR 78–144) (table 4, online supplemental figure 2). Patients with PHECG were also more likely to receive reperfusion within 90 min of the call for help (24.5% vs 21.8%, aOR 1.18, 95% CI 1.11 to 1.25).

**Table 4:** Time to reperfusion in STEMI patients undergoing reperfusion

	PHECG (n=109 998)		No PHECG (n=7659)		Comparison	95% CI
	Median	IQR	Median	IQR	Adjusted HR	
Minutes from call for help to receiving reperfusion	112	(93, 137)	122	(94, 166)	1.49	1.45 to 1.52
Minutes from arrival of ambulance to receiving reperfusion	97	(78, 120)	103	(78, 144)	1.38	1.35 to 1.42
Minutes from hospital door to receiving reperfusion	40	(28, 59)	45	(30, 82)	1.39	1.36 to 1.43
	N	%	N	%	Adjusted OR	95% CI
Received reperfusion within 90 min of call for help.	25 896/105 828	24.5%	1430/6563	21.8%	1.18	1.11 to 1.25

ORs and HRs are adjusted for age (greater than 75 vs not), sex, ethnicity (Caucasian vs Asian vs other ethnic groups), prior myocardial infarction, prior coronary artery bypass surgery; diabetes, prior heart failure; chronic kidney disease; prior stroke/cerebrovascular disease.

Counts may not sum to overall values due to missing data.

PHECG, prehospital ECG; STEMI, ST-elevation myocardial infarction.

## Discussion

International clinical guidelines for the management of ACS extant towards the end of our study supported performance of PHECG by EMS personnel.^1^ Current guidelines recommend that EMS ambulances are equipped with ECG recorders and that PHECG should be performed as soon as possible when ACS is suspected to determine the initial treatment pathway and inform conveyance decisions.^11^ Those with STEMI and those with continuing ischaemic symptoms without ST-segment elevation would normally be transported to hospitals providing immediate coronary interventions. Further, PHECG performance and interpretation consistently appears among descriptors of good quality care of both STEMI and nSTEMI.^2 12^

The proportion of patients receiving PHECG increased from 74.2% in 2010 to 85.0% in 2017. This continues the trend we first reported in the ‘thrombolysis-era’, from 51% in 2005 to 64% in 2009, prior to implementation of the existing pPCI approach to STEMI management.^6^ However, 20.3% did not receive PHECG during this study. Patients who did not receive PHECG were older, female and likely to have comorbidities; those receiving PHECG were more likely to have prior PCI, dyslipidaemia and be smokers.

Although consistent with our previous work, it is not clear from MINAP data why some patients are less likely to receive PHECG.^6^ It may be that symptoms experienced and/or reported more frequently by females and by people with these comorbidities do not ‘trigger’ the performance of an ECG. In a German registry covering the same period as our study, patients with diabetes experiencing their first MI, particularly those older than 55 years, were less likely than those without diabetes to experience the type of chest pain historically considered ‘typical’ of MI presentation, and more likely to experience breathlessness; though much of this difference was attenuated when accounting for co-existence of poor ventricular function and renal impairment.^13^ Interviews with diabetes patients hospitalised following acute MI revealed that while the majority experienced chest pain during the event, their overall experience did not live up to their expectation of a ‘heart attack’ and they interpreted it as a hypoglycaemic event—which, if accepted by the attending EMS, may reduce the likelihood of PHECG.^14^

Our findings are consistent with others in relation to gender disparities in prehospital ACS care.^15 16^ A systematic review of the signs and symptoms of MI reported that both sexes experienced chest pain, but females more often presented with other symptoms such as nausea, vomiting and breathlessness, and prodromes of vague sleep disturbance and fatigue—which may distract the attending paramedic from the need to perform an ECG.^17^ Semistructured interviews of paramedics revealed concerns about gender-concordance (or lack thereof) between practitioner and patient, with male paramedics expressing hesitancy in exposing a woman’s chest.^18^ Safety investigations into the training and competence of British paramedics in PHECG extend these concerns to include religious and cultural considerations and difficulties removing electrodes from older patients’ skin.^19^

We found systolic blood pressure at hospital admission was marginally lower in patients with PHECG (135 mm Hg) than those without (137 mm Hg), as was heart rate on admission (77 beats/min vs 80). If these differences indicate what was present before any EMS intervention, they suggest PHECG is offered to lower risk patients with respect to heart rate, but higher risk patients with respect to systolic blood pressure. This suggests it is unlikely EMS personnel simply ‘scoop and run’ with more ill-looking patients.

Current quality indicators for ACS management include unadjusted all-cause in-hospital mortality as a single expression of outcome.^12^ We chose to present the previously recommended quality indicator—adjusted 30-day mortality and found that a PHECG recorded in MINAP is associated with reduced 30-day and 1-year mortality for both STEMI and nSTEMI patients, regardless of reperfusion strategy for STEMI patients. If PHECGs were recorded at the 2017 rate for the entire study period, an estimated 17 686 additional patients would have received one. With a 30-day mortality of 7.1% in patients receiving PHECG compared with 10.9% in patients without PHECG, we would expect approximately 672 fewer deaths within 30 days over the study period. If, instead, patients received PHECG at the highest level recorded in a single EMS in a single year, an estimated 53 679 additional patients would have received one, corresponding to approximately 2040 fewer deaths within 30 days over the study period. This demonstrates the importance of addressing the variations in care reported in the literature. Simms *et al* found missed opportunities were significantly associated with mortality, and prehospital missed opportunities predicted later failures associated with adverse outcomes.^20^

A lower proportion of STEMI patients with PHECG received thrombolysis compared with those without. However, it is not clear whether PHECG influenced the type of reperfusion, or if there is a mutual confounding factor (eg, incident year).

The median time from ambulance arrival to hospital was longer for patients with PHECG recorded, consistent with meta-analysis findings.^21^ However, STEMI patients with PHECG were more likely to receive reperfusion, and more timely reperfusion; the median time from call to reperfusion was 10 min faster in patients with PHECG, widening to 29 min at the upper quartile.

Less than a quarter of STEMI patients received reperfusion within 90 min of their call for help, only marginally higher in patients with PHECG than without (24% vs 21%). This may suggest that the 90 min European Society of Cardiology quality indicator target is too ambitious, and the recent guideline of 120 min is more realistic.^11 12^

The focus of PHECG is identification of ACS patients with STEMI, whose outcomes can be improved by rapid reperfusion via pPCI. It is less clear by what mechanism PHECG is associated with better 30-day survival in those with a final diagnosis of nSTEMI for whom routine immediate reperfusion is not mandated. However, we note the 2023 ECS guidelines recommend almost all nSTEMI patients receive angiography, and some within 24 hours, or even 2 hours, based on risk.^11^

### Strengths and limitations

The main strengths of this study are the very large population and its multifaceted, multicentre approach to understanding barriers and facilitators to PHECG.

The main limitation of the study is its observational, cross-sectional nature, which precludes inferring causal relationships. Although findings are consistent with our previous work, this analysis used final diagnosis to determine patient eligibility, whereas that work used initial diagnosis.^5^ This does not impact generalisability, but does mean the two works are not fully comparable. This may partly explain the difference in proportion of PHECGs reported between 2009 in the previous work and 2010 herein. Age was dichotomised to maximise comparability with the previous study, where treating it as a continuous variable would otherwise be preferable. We did not adjust for reperfusion in STEMI patients on the same basis; although subgroup analyses for STEMI patients with/without reperfusion are presented and suggest this would be an important factor for future research. While we investigated time to reperfusion for STEMI patients, we did not investigate time to angiography for nSTEMI patients, despite 2023 ACS guidelines recommending angiography during hospitalisation.^11^ MINAP does not record ACS symptoms or prehospital haemodynamic measurements; therefore, we were unable to investigate any association with PHECG provision. Finally, we are unable to ascertain the extent to which patients under EMS care received PHECG that was not recorded in MINAP.

## Conclusions

Patients receiving PHECG were younger, with fewer comorbidities, than those without PHECG. Although the proportion of ACS patients receiving PHECG was fairly high, and increased during the study period, systematic inequalities in administration associated with important differences in processes and outcomes of care persist. EMS providers must address these variations in care to avoid increasing health inequalities.

## Supplementary material

10.1136/heartjnl-2025-325780 online supplemental file 1

## Data Availability

Data may be obtained from a third party and are not publicly available.
