# Supplementary material for: Prehospital 12-lead ECG and outcomes in acute coronary syndrome
Source: Heart. 2025 Sep 10;112(15):e325780. doi: 10.1136/heartjnl-2025-325780 (PMC13479262; doi:10.1136/heartjnl-2025-325780)
Supplement: Supplementary file 1 [file heartjnl-112-15-s001.docx]

# Supplementary Material

### Supplementary Figure 1: Distribution of times from call for help to arrival at hospital by receipt of PHECG

### Supplementary Table 1: Use of reperfusion in patients with STEMI

|  | | **Reperfusion**  (n=117,657) | | **No reperfusion**  (n=26,127) | | **Adjusted Odds Ratio** | **95% Confidence Interval** |
| --- | --- | --- | --- | --- | --- | --- | --- |
|  |  | **n** | **%** | **n** | **%** |  |  |
| PHECG recorded | | 109,998  /117,657 | 93.5% | 19,873  /19873 | 76.1% | 4.37 | (4.20 – 4.54) |
| Older than 75 years | | 27,968  /117,625 | 23.8% | 13,821  /26,115 | 52.9% | 0.33 | (0.32 – 0.34) |
| Female | | 31,096  /116,522 | 26.7% | 10,923  /26,038 | 42.0% | 0.68 | (0.66 – 0.70_ |
| Prior myocardial infarction recorded | | 10,559  /108,256 | 9.8% | 3,770  /24,797 | 15.2% | 0.76 | (0.73 – 0.80) |
| Prior coronary artery bypass grafts | | 2,120  /107,846 | 2.0% | 1,031  /24,665 | 4.2% | 0.65 | (0.59 – 0.71) |
| Diabetes mellitus | | 16,268  /111,650 | 14.6% | 5,255  /25,326 | 20.7% | 0.74 | (0.71 – 0.77) |
| Prior heart failure | | 1,561  /107,586 | 1.5% | 1,285  /24,572 | 5.2% | 0.45 | (0.42 – 0.49) |
| Year of incident | 2010 | 15,110 | 12.8% | 3,687 | 14.1% | 1.01^a^ | (1.00 – 2.78)^a^ |
|  | 2011 | 14,604 | 12.4% | 3,424 | 13.1% |  |  |
|  | 2012 | 15,327 | 13.0% | 3,514 | 13.4% |  |  |
|  | 2013 | 14,642 | 12.4% | 3,374 | 12.9% |  |  |
|  | 2014 | 14,580 | 12.4% | 3,279 | 12.6% |  |  |
|  | 2015 | 14,378 | 12.2% | 2,977 | 11.4% |  |  |
|  | 2016 | 14,072 | 12.0% | 2,897 | 11.1% |  |  |
|  | 2017 | 14,944 | 12.7% | 2,975 | 11.4% |  |  |

*Odds ratios are adjusted for PHECG use, age (greater than 75 vs not), sex, ethnicity (Caucasian vs Asian vs other ethnic groups), receipt of aspirin, raised cardiac markers, chronic kidney disease, diabetes, prior stroke/cerebrovascular disease, and prior CHF.*

*^a^ Adjusted odds ratio per year since 2010.*

### Supplementary Figure 2: Distribution of times for STEMI patients receiving reperfusion by receipt of PHECG
